# Antidiabetic treatment patterns and specialty care utilization among patients with type 2 diabetes and cardiovascular disease

**DOI:** 10.1186/s12933-018-0699-7

**Published:** 2018-04-10

**Authors:** Kevin M. Pantalone, Anita D. Misra-Hebert, Todd M. Hobbs, Xinge Ji, Sheldon X. Kong, Alex Milinovich, Wayne Weng, Janine Bauman, Rahul Ganguly, Bartolome Burguera, Michael W. Kattan, Robert S. Zimmerman

**Affiliations:** 1 Department of Endocrinology, Endocrinology and Metabolism Institute, Cleveland Clinic, 9500 Euclid Avenue, Desk F-20, Cleveland, OH USA; 2 Medicine Institute, Cleveland Clinic, Cleveland, OH USA; 3 Quantitative Health Sciences, Cleveland Clinic, Cleveland, OH USA; 4 Novo Nordisk Inc., Plainsboro, NJ USA; 5 Health Economics and Outcomes Research, Novo Nordisk Inc., Plainsboro, NJ USA; 6 Bariatric and Metabolic Institute, Cleveland Clinic, Cleveland, OH USA

**Keywords:** Cardiovascular disease, Type 2 diabetes, Population health, Antidiabetic therapies

## Abstract

**Background:**

To evaluate real-world patient characteristics, medication use, and health care utilization patterns in patients with type 2 diabetes with established cardiovascular disease (CVD).

**Methods:**

Cross-sectional analysis of patients with type 2 diabetes seen at Cleveland Clinic from 2005 to 2016, divided into two cohorts: with-CVD and without-CVD. Patient demographics and antidiabetic medications were recorded in December 2016; department encounters included all visits from 1/1/2016 to 12/31/2016. Comorbidity burden was assessed by the diabetes complications severity index (DCSI) score.

**Results:**

Of 95,569 patients with type 2 diabetes, 40,910 (42.8%) were identified as having established CVD. Patients with CVD vs. those without were older (median age 69.1 vs. 58.2 years), predominantly male (53.8% vs. 42.6%), and more likely to have Medicare insurance (69.4% vs. 35.3%). The with-CVD cohort had a higher proportion of patients with a DCSI score ≥ 3 than the without-CVD cohort (65.0% vs. 10.3%). Utilization rates of glucagon-like peptide-1 receptor agonists and sodium–glucose co-transporter-2 inhibitors were low in both with-CVD (4.1 and 2.5%) and without-CVD cohorts (5.4 and 4.1%), respectively. The majority of patient visits (75%) were seen by a primary care provider. During the 1-year observation period, 81.9 and 62.0% of patients with type 2 diabetes and CVD were not seen by endocrinology or cardiology, respectively.

**Conclusions:**

These data indicated underutilization of specialists and antidiabetic medications reported to confer CV benefit in patients with type 2 diabetes and CVD. The impact of recently updated guidelines and cardiovascular outcome trial results on management patterns in such patients remains to be seen.

## Background

Type 2 diabetes is a significant risk factor in the development of cardiovascular disease (CVD). The Framingham heart study reported diabetes to be associated with a two to fourfold increased risk of myocardial infarction (MI), congestive heart failure, peripheral arterial disease, stroke, and increased mortality [1–3]. In addition, having diabetes and established CVD substantially decreases life expectancy [4].

Thus far, randomized controlled trials have failed to conclusively demonstrate that more aggressive glycemic control in high-risk cardiovascular (CV) patients translates into a reduction in CV-risk or mortality [5–7]. In the action to control cardiovascular risk in diabetes (ACCORD) trial [6], the use of intensive therapy to target normal glycated hemoglobin (A1C) levels for 3.5 years actually increased mortality. However, recently published cardiovascular outcome trials (CVOTs) have reported CV risk reduction with two sodium–glucose co-transporter-2 inhibitors, (SGLT-2i; empagliflozin [8], FDA-approved December, 2016 and canagliflozin [9], FDA-approved March 2013), and two glucagon-like peptide 1 receptor agonists, (GLP-1RA; liraglutide [10], FDA-approved January 2010 and semaglutide [11], FDA-approved December 2017). It is unlikely that the observed differences in CV risk with these active medications vs. comparator (placebo) groups (given in addition to other standard diabetes care) can be attributed to tighter glycemic control alone, as the differences in glycemic control (A1C) between study arms were ≤ 1% in all four trials. Rather, these observations suggest that there may be a unique mechanism or property inherent to these particular agents that confer this CV risk reduction. A recent meta-analysis that included the liraglutide and semaglutide CVOT data [10, 11] reported that GLP-1 RA therapies reduced the risk of CV mortality compared with placebo (relative risk = 0.84, 95% CI 0.73–0.97) [12].

It would therefore be paramount that patients with type 2 diabetes and established CVD be considered to receive these therapies in order to reduce their residual risk of adverse CV outcomes which remains even after aggressive treatment of other existing CV risk factors. Indeed, after results of the empagliflozin and liraglutide cardiovascular outcome trials became available and debated, the American Diabetes Association (ADA) included in the standards of medical care in diabetes-2017 the recommendation that in patients with longstanding sub-optimally controlled type 2 diabetes and established atherosclerotic CVD, empagliflozin or liraglutide should be considered as they have been shown to reduce CV and all-cause mortality when added to the standard of care [13].

The impact of new diabetes guidelines on patient management in general and CV outcomes specifically will certainly be a subject of great interest over coming years. As a baseline to allow for future evaluation of this new recommendation, we used a large electronic health records database to assess real-world treatment patterns among patients with type 2 diabetes and CVD over a time period shortly preceding the release of the updated diabetes management guidelines.

## Methods

### Dataset and patient inclusion

The electronic health record (EHR) system at Cleveland Clinic (Ohio and Florida) was used to create a cross-sectional summary of patients with type 2 diabetes in 2016. The study cohort included patients with type 2 diabetes identified from 2005 to 2016 via a modified version of the EMERGE algorithm published by Kho et al. [14] international classification of diseases, ninth revision (ICD-9) codes of 250.x0 or 250.x2 or ICD-10 codes of E11.xx were used to identify patients with type 2 diabetes. Patients with an ICD-9 code specific for type 1 diabetes (250.x1, 250.x3) at any time were excluded from the cohort. Ketoacidosis codes (250.10 and 250.12) were classified as type 1 diabetes. The algorithm was used to calculate the earliest date when a patient record contained any of the following combinations: type 2 diabetes code and type 2 diabetes medication; type 2 diabetes code and abnormal glucose; type 2 diabetes code recorded twice and outpatient insulin prescription; type 2 diabetes medication and abnormal glucose; or insulin preceded by type 2 diabetes medication. Abnormal glucose was defined according to ADA criteria (fasting blood glucose [BG] ≥ 126 mg/dL, A1C ≥ 6.5%, or random BG ≥ 200 mg/dL). The earliest date that any of the five conditions was met was documented as the date on which the patient first met the criteria for type 2 diabetes. Patients had to be ≥ 18 years of age as of January 1, 2016, and had to have at least one visit with family medicine, internal medicine, or endocrinology between January 1, 2015 and December 31, 2016. Data available within the EHR as of December 31, 2016 were utilized for the analysis.

### Study cohorts—patients with and without established CVD

Patients were subsequently stratified into two categories, those with established CVD (with-CVD) and those without established CVD (without-CVD), using the definitions outlined by the ADA 2017 guidelines [15]. Established atherosclerotic CVD (hereafter, “CVD”), per the ADA 2017 guidelines, was defined as follows: Acute coronary syndrome, history of myocardial infarction, stable or unstable angina, coronary or other arterial revascularization, stroke, transient ischemic attack, or peripheral arterial disease presumed to be of atherosclerotic origin. These forms of established CVD were recognized by documentation of corresponding ICD-9 and ICD-10 codes within the EHR (see Appendix Table 4 for detailed list).

The subset of patients without established CVD was further stratified based on the number of other recognized risk factors for CVD (0, 1, or ≥ 2). Those risk factors included: female > 55 years of age, male > 45 years of age, family history of CVD, tobacco use-current, LDL > 130 mg/dL, hypertension diagnosis, body mass index ≥ 30 kg/m^2^, age ≥ 60 years and albuminuria (urine albumin above upper limit of normal or albumin: creatinine ratio > 30) or age ≥ 50 years with estimated glomerular filtration rate (eGFR) < 60 mL/min/1.73 m^2^), based on outpatient creatinine measurements only, calculated using the chronic kidney disease epidemiology collaboration (CKD-EPI) equation [16, 17]. For the eGFR determination, patients must have had two values < 60 that were at least 90 days apart, with no normal values (eGFR ≥ 60) in between.

### Variables of interest

Patient age, race/ethnicity, gender, insurance status and median household income were recorded. Income was defined as the 2011–2015 5-year estimates of median household income at the block group level obtained from the American community survey [18] conducted by the US Census Bureau. Comorbidities of hypertension and hyperlipidemia and diabetes complications (retinopathy, nephropathy, neuropathy, cerebrovascular disease, cardiovascular disease, peripheral vascular disease) were captured by relevant ICD-9/ICD-10 codes.

For the purposes of this study, medication utilization data were recorded based on medications documented in the patient’s EHR at the end of 2016. A given medication had to be active on the patient’s medication list for at least 3 months in order to be included. Medication classes included the following: biguanide (metformin), sulfonylurea (SFU), thiazolidinedione, dipeptidyl-peptidase-4 inhibitor (DPP-4i), alpha-glucosidase inhibitor, glucagon-like peptide-1 receptor agonist (GLP-1RA), sodium–glucose co-transporter-2 inhibitor (SGLT-2i), and insulin (basal, bolus, or mixed formulation).

Healthcare utilization patterns were assessed by identifying completed appointments in the following departments: internal medicine/family medicine, endocrinology, and cardiology. The number of visits with each department were categorized (0, 1–3, ≥ 4), and then stratified by presence or absence of established CVD. Encounter department visits were recorded as a sum of all encounters from January 1, 2016 to December 31, 2016.

The diabetes complications severity index (DCSI) score was calculated for all patients based on comorbidities identified by ICD-9 codes [19]. The patients were then categorized as a having a score of 0, 1–2, or ≥ 3 and subsequently stratified by CVD status (established CVD or no-established CVD) to assess the comorbidity burden in each population.

### Data analysis

Characteristics were measured using counts with percentages for the categorical variables and medians with interquartile range (25th, 75th percentile) for the continuous variables. Categorical variables were evaluated for association using the Chi squared test; continuous variables were tested using the Mann–Whitney U test. P < 0.05 were considered statistically significant.

## Results

### Distribution of cardiovascular risk level

A total of 95,569 patients were identified for this analysis: 40,910 (42.8%) with-CVD and 54,659 (57.2%) without. Among those without established CV disease, 50,362 (92.1%) had ≥ 2 risk factors for CVD, 3664 (6.8%) had 1 risk factor for CVD, and 633 (1.1%) had no identifiable risk factors for CV disease, other than established type 2 diabetes.

### Demographics of population

Patient demographics and baseline clinical characteristics, stratified by CVD status, are summarized in Table 1. Patients with established CVD were observed to be older, with a median (IQR) age of 69.1 (60.8, 77.3) years compared to those without established CVD, median (IQR) age 58.2 (48.2, 67.0) years. Racial distribution (White, Black, other) was generally numerically similar among the cohorts with and without established CVD, despite testing as significantly different. A higher percentage of males was observed in patients with established CVD (53.8% vs. 42.6%). Medicare was the most common form of insurance in patients with established CVD (69.4%), whereas commercial insurance was the most common form of coverage in patients without established CVD (46.9%). The median income (US dollars, IQR) was lower in patients with established CVD vs. those without established CVD; $51,700 (41,100, 66,900) vs. $54,300 (41,800, 67,400). The with-CVD cohort had significantly higher prevalence of hypertension and hyperlipidemia, and also of the DCSI component complications retinopathy, nephropathy, neuropathy, cerebrovascular, cardiovascular, and peripheral vascular disease. All of the above cited comparisons between patients with-CVD vs. those without-CVD were statistically significant (P < 0.001).

**Table 1:** Demographic characteristics of real-world 2016 type 2 diabetes population, stratified by cardiovascular disease (CVD) status

Variable	Missing n (%)	No established CVD N = 54,659	Established CVD N = 40,910	P value*
Age, median (IQR)		58.2 (48.2, 67.0)	69.1 (60.8, 77.3)	< 0.001
Race, n (%)	2677 (3%)			< 0.001
White		40,779 (77%)	31,465 (78.8%)	
Black		9892 (18.7%)	7265 (18.2%)	
Other		2308 (4.4%)	1183 (3%)	
Gender, n (%)	3 (0%)			< 0.001
Female		31,385 (57.4%)	18,916 (46.2%)	
Male		23,273 (42.6%)	21,992 (53.8%)	
Insurance, n (%)	8602 (9%)			< 0.001
Commercial		22,656 (46.9%)	7964 (20.6%)	
Employee health insurance		3108 (6.4%)	1017 (2.6%)	
Managed care		993 (2.1%)	383 (1%)	
Medicaid		4277 (8.9%)	2380 (6.2%)	
Medicare		17,032 (35.3%)	26,840 (69.4%)	
Military personnel		223 (0.5%)	93 (0.2%)	
Median zip income (US$, thousands)	1484 (2%)	54.3 (41.8, 67.4)	51.7 (41.1, 66.9)	< 0.001
HbA1c (%), median (IQR)		6.7 (6.1, 7.6)	6.6 (6.1, 7.4)	0.002
Hypertension, n (%)		40,666 (74.4%)	38,267 (93.5%)	< 0.001
Hyperlipidemia, n (%)		36,367 (66.5%)	34,653 (84.7%)	< 0.001
Current smoker, n (%)		6264 (11.5%)	4313 (10.5%)	< 0.001
Body mass index ≥ 30 kg/m^2^, n (%)		38,183 (69.9%)	26,936 (65.8%)	< 0.001
Family history of CV disease, n (%)		36,064 (66.0%)	28,702 (70.2%)	< 0.001
DCSI score				< 0.001
0		32,825 (60.1%)	6166 (15.1%)	
1–2		16,182 (29.6%)	8149 (19.9%)	
≥ 3		5652 (10.3%)	26,595 (65%)	
Diabetes complications^a^				
Retinopathy				< 0.001
Not present		50,771 (92.9%)	35,445 (86.6%)	
Abnormal		3068 (5.6%)	4126 (10.1%)	
Severe abnormal		820 (1.5%)	1339 (3.3%)	
Nephropathy				< 0.001
Not present		38,131 (69.8%)	14,027 (34.3%)	
Abnormal		10,530 (19.3%)	13,937 (34.1%)	
Severe abnormal		5998 (11%)	12,946 (31.6%)	
Neuropathy				< 0.001
Not present		41,536 (76%)	24,485 (59.9%)	
Abnormal		13,123 (24%)	16,425 (40.1%)	
Cerebrovascular				< 0.001
Not present		54,657 (100%)	27,587 (67.4%)	
Abnormal		0 (0%)	1681 (4.1%)	
Severe abnormal		2 (0%)	11,642 (28.5%)	
Cardiovascular^b^				< 0.001
Not present		52,079 (95.3%)	14,771 (36.1%)	
Abnormal		42 (0.1%)	10,250 (25.1%)	
Severe abnormal		2538 (4.6%)	15,889 (38.8%)	
Peripheral vascular disease				< 0.001
Not present		54,039 (98.9%)	30,920 (75.6%)	
Abnormal		577 (1.1%)	9203 (22.5%)	
Severe abnormal		43 (0.1%)	787 (1.9%)	

*CVD* cardiovascular disease, *DCSI* diabetes complications severity index, *IQR* interquartile range

*Categorical variables were evaluated for association using the Chi squared test; continuous variables were tested using the Mann–Whitney U test

^a^DCSI complications identified using ICD-9 codes as outlined by Young et al. [19]. For each complication, scale 0 = no abnormality, 1 = some abnormality, 2 = severe abnormality, as classified by Young et al.

^b^Category reflects DCSI-specific cardiovascular complications: atherosclerosis; other ischemic heart disease; angina pectoris; other chronic ischemic heart disease; myocardial infarction; ventricular fibrillation, arrest; atrial fibrillation, arrest; other atherosclerotic cardiovascular disease; old myocardial infarction; heart failure; atherosclerosis, severe; aortic aneurysm/dissection

### Diabetes complications severity index scores

The with-CVD cohort contained a higher percentage of patients with a DCSI score ≥ 3 (Table 1). The distribution of DCSI score categories 0, 1–2, and ≥ 3 in the with-CVD cohort was 15.1, 19.9, and 65.0%, respectively, compared with the without-CVD cohort in which the distribution was 60.1, 29.6, and 10.3%, respectively (P < 0.001).

### Antidiabetic medication treatment patterns stratified by CV disease status

Table 2 provides a summary of antidiabetic medication treatment patterns stratified by CVD status. In patients with established CVD (N = 40,910), 58.1% were prescribed an oral anti-diabetes drug (OAD); 36.4% were prescribed 1 OAD, 16.1% were prescribed two OADs, and 5.6% were prescribed ≥ 3 OADs. Among patients without established CVD (N = 54,659), the corresponding numbers were 67.1, 42.4, 17.4, and 7.3%. Insulin utilization among those with established CVD was 18.3% vs. 11.4% in those without established CVD (all P < 0.001).

**Table 2:** Antidiabetic medication treatment patterns stratified by cardiovascular disease (CVD) status

Medication^a^	No established CVD N = 54,659	Established CVD N = 40,910	P value^†^
No OAD	17,984 (32.9%)	17,137 (41.9%)	< 0.001
OAD	36,675 (67.1%)	23,773 (58.1%)	
1 OAD	23,166 (63.2%)	14,889 (62.6%)	< 0.001
2 OAD	9540 (26.0%)	6574 (27.7%)	< 0.001
≥ 3 OAD	3969 (10.8%)	2310 (9.7%)	< 0.001
Insulin	6211 (11.4%)	7472 (18.3%)	< 0.001
GLP-1RA	2978 (5.4%)	1685 (4.1%)	< 0.001
Liraglutide	1683 (3.1%)	916 (2.2%)	< 0.001
SGLT-2i	2265 (4.1%)	1042 (2.5%)	< 0.001
Empagliflozin	462 (0.8%)	209 (0.5%)	< 0.001
Canagliflozin	1348 (2.5%)	691 (1.7%)	< 0.001
Other ADD	1101 (2%)	853 (2.1%)	0.444

OADs: biguanide (metformin), sulfonylurea, thiazolidinedione, dipeptidyl-peptidase-4 inhibitor, alpha-glucosidase inhibitor, sodium–glucose co-transporter-2 inhibitor

Other ADD-other antidiabetic drug: pramlintide, name brand bromocriptine (Cycloset^®^), colesevelam, nateglinide or repaglinide

*ADD* antidiabetic drug, *CVD* cardiovascular disease, *GLP-1RA* glucagon-like peptide-1 receptor agonist, *OAD* oral antidiabetic drug

^**†**^Categorical variables were evaluated for association using the Chi squared test; continuous variables were tested using the Mann–Whitney U test

^a^Medication categories are not mutually exclusive, and patients could be represented in more than one category (“OAD” sub-categories by number of OADs are mutually exclusive within that category)

In patients with established CVD, 4.1% were prescribed GLP-1RA therapy, vs. 5.4% in those without established CVD. Specifically, liraglutide utilization was 2.2% among those with established CVD vs. 3.1% in those without established CVD. SGLT-2i therapy was prescribed to 2.5% of patients with established CVD vs. 4.1% of patients without established CVD. Empagliflozin and canagliflozin were prescribed in 0.5 and 1.7% of patients with established CVD, as compared with 0.8 and 2.5% in the population of patients without established CVD, respectively (all P < 0.001). The use of other antidiabetic drugs was similar in those with (2.1%) and without (2.0%) established CVD (P = 0.444). Other antidiabetic drugs included pramlintide, timed-release bromocriptine (Cycloset^®^), colesevelam, nateglinide, or repaglinide.

### Health care utilization patterns

Between January 1, 2016 and December 31, 2016, 25.8% of patients with type 2 diabetes and established CVD did not have an appointment with a primary care provider (PCP, internal medicine or family medicine), 39.8% were seen 1–3 times, and 34.5% were seen ≥ 4 times (Table 3). In those without established CVD, 25.0% were not seen by a PCP, 49.5% were seen 1–3 times, and 25.5% ≥ 4 times.

**Table 3:** Health Care Utilization Patterns Stratified by Cardiovascular Disease (CVD) Status

Encounter department	No established CVD N = 54,659	Established CVD N = 40,910	P value*
Internal/family medicine, n (%)			< 0.001
0 visits	13,639 (25.0)	10,536 (25.8)	
1–3 visits	27,083 (49.5)	16,266 (39.8)	
≥ 4 visits	13,937 (25.5)	14,108 (34.5)	
Endocrinology, n (%)			0.022
0 visits	44,405 (81.2)	33,517 (81.9)	
1–3 visits	8911 (16.3)	6405 (15.7)	
≥ 4 visits	1343 (2.5)	988 (2.4)	
Cardiology, n (%)			< 0.001
0 visits	48,926 (89.5)	25,377 (62.0)	
1–3 visits	5245 (9.6)	11,801 (28.8)	
≥ 4 visits	488 (0.9)	3732 (9.1)	

*Categorical variables were evaluated for association using the Chi squared test; continuous variables were tested using the Mann–Whitney U test

Among patients with established CVD, 81.9% did not have an appointment with endocrinology, 15.7% were seen 1–3 times, and 2.4% were seen ≥ 4 times. In those without established CVD, 81.2% did not have an appointment with endocrinology, 16.3% 1–3 times, and 2.5% ≥ 4 times.

In patients with established CVD, 62.0% did not have an appointment with cardiology, 28.8% were seen 1–3 times, and 9.1% ≥ 4 times. In patients without established CVD, 89.5% did not have an appointment with cardiology, 9.6% were seen by cardiology 1–3 times, and 0.9% ≥ 4 times. All comparisons, with-CVD versus without-CVD, were significant (P < 0.001).

## Discussion

In this real-world population of patients with type 2 diabetes managed at Cleveland Clinic, the subgroup of patients with established CVD was on average older, contained a higher percentage of males, and had a larger proportion of Medicare-insured patients, when compared to the subgroup without established CVD. The distribution of race/ethnicity was similar between the two groups, with the majority of patients being identified as White. Patients with established CVD were observed to have a higher comorbidity burden, per the observed percentages of patients with a DCSI score ≥ 3, when compared to patients without established CVD. While these observations are not surprising, they provide context to the treatment and healthcare utilization patterns that were observed in our population of patients with type 2 diabetes.

To the best of our knowledge, this is the first real-world assessment of T2D treatment patterns in patients with concomitant CVD in a large system-wide EHR. The rather low utilization rates of therapeutic agents which have been recently reported to reduce CV risk among patients with type 2 diabetes highlight that these therapies were not being widely used during 2016 in patients with established CVD, which is the population most likely to benefit. In patients with type 2 diabetes and established CVD, the utilization rates of these medications were very low: empagliflozin (0.5%), canagliflozin (1.7%), liraglutide (2.2%). Given that the CVOTs with these three medications were only published in 2015, [8] 2017, [9] and 2016, [10] respectively, the findings were likely too recent to have achieved broad adaptation and acceptance by prescribers during the study period of 2016. Other potential reasons for low utilization rates of these specific therapies include our observation that the population of patients with established CVD were older, had a higher comorbidity burden, and/or were more likely to have Medicare coverage. Both older age and higher comorbidity burden could make use of the newer antidiabetic therapies more challenging for reasons that may include living on a fixed income, having comorbidities that may serve as a contraindication to therapy (i.e., CKD for SGLT-2s), etc. Nearly 70% of patients with established CVD were covered by Medicare, which makes access to these newer therapies more challenging because of “donut-hole” coverage gaps and the inability to use medication co-pay cards, issues that generally do not affect commercially insured patients. In addition, insurance formularies may give preference to alternative classes of medications (i.e., SFU, or DPP-4i), or other specific agents within a medication class (i.e., exenatide rather than liraglutide). However, even in the population of patients without established CVD, which was observed to contain younger individuals with a lower comorbidity burden, and more patients covered by commercial insurance, the utilization rates of the GLP-1RA and SGLT-2i classes of medications were still observed to be rather low overall. These patients may also have challenges in attaining the newer classes of antidiabetic therapies (high-deductible health care plans, medication cost, etc.).

The treatment landscape may change dramatically in the near future should clinicians begin to implement therapies consistent with the recently published CVOTs with empagliflozin, [8] canagliflozin, [9] liraglutide, [10] and semaglutide, [11] as well as current disease management guidelines. The most recent American association of clinical endocrinologists (AACE) guidelines [20] give preference in their treatment algorithm to the antidiabetic therapies associated with weight loss and/or a low risk of hypoglycemia (i.e., GLP-1RA and SGLT-2i), and the American Diabetes Association’s pharmacologic approaches to glycemic treatment guidelines [13] suggest that in patients with longstanding sub-optimally controlled type 2 diabetes and established atherosclerotic CVD, empagliflozin or liraglutide should be considered. As having diabetes and a prior history of MI or stroke has been reported to reduce life expectancy by 12 years vs. those without diabetes, [4] it is imperative that clinicians do everything within their power to help lower this very high-risk populations’ residual risk of both CV events and/or death. This includes not only implementing the current standard-of-care in blood pressure and lipid management, but also choosing specific therapies to improve glycemic control which have been demonstrated to also afford CV benefit. Evidence continues to mount that good glycemic control alone is not enough to produce improved CV outcomes in patients with T2D and therapy choices need to take extra-glycemic CV risks and benefits into account [21].

Patients with established CVD, who were observed to be older and have higher comorbidity burden (DCSI score ≥ 3), were less likely to be receiving only OADs when compared to those without established CVD. This is largely because insulin (alone or in combination with OADs) was observed to be used more frequently in this population (18.3% vs. 11.4% of patients without established CVD). Certainly, there are many patients with CVD that require insulin therapy for various reasons: progression of longstanding diabetes; failure to achieve glycemic control by other means (i.e., OAD); or because of comorbidities (i.e., CKD) that make insulin therapy a preferred therapeutic choice. However, in patients with established CVD who do not have a contraindication to GLP-1RA or SGLT-2i, the results of the recently published CVOTs would support the use of liraglutide, empagliflozin, or canagliflozin. In addition, the results of the liraglutide effect and action in diabetes: evaluation of cardiovascular outcome results (LEADER) trial [10] would support the use of liraglutide vs. insulin as the preferred injectable treatment in the population of patients with established CVD (if glycemic control and/or comorbidity profile allowed for its use). Interestingly, GLP-1RAs and SGLT-2i agents were being used in significantly greater proportions of patients without established CVD compared with patients having established CVD. Again, these findings reflect the treatment environment prior to or just slightly overlapping recent indications for medications with potential CVD benefits.

In the current study, specialty care among the cohort of patients with established CVD was observed to be rather low, particularly by the endocrinology specialty; in fact, 81.9% of these patients did not have an appointment with the department of endocrinology within 1 year prior to December 31, 2016. This may be contributing to the low rates of SGLT-2i and GLP-1RA therapy utilization observed not only in the population of patients with established CVD, but also in those without established CVD, where the likelihood of not being seen by an endocrinologist was essentially the same (81.2%). We suspect this may be due in large part to access/availability limitations for endocrinology specialists which results in only the most “severe cases” of diabetes referred to endocrinology, a scenario likely not unique to Cleveland Clinic, unfortunately. Department of cardiology appointments were somewhat more prevalent in patients with established CVD, yet, 62.0% of such patients did not have one visit with this specialty within the last 1 year prior to December 31st, 2016. While it is possible that some of these patients may have obtained specialty care outside of Cleveland Clinic, this percentage would be expected to be low given Cleveland Clinic is a tertiary care center where patients are referred to receive specialty care. Patients with established CVD may be more likely to receive the therapies that have been demonstrated to confer CV benefit if they are seen by either endocrinology or cardiology specialists. As endocrinologists would be expected to be the most familiar with these newer therapies, the most recent diabetes management guidelines, and the results of CVOTs pertaining to antidiabetic therapies, it would seem important that patients with established CVD and type 2 diabetes be referred to endocrinology for specialty care. While cardiologists may not be likely to prescribe these antidiabetic medications directly, visits with this specialty may at least result in conversations with patients about the cardiovascular implications of diabetes and the potential utility of SGLT-2i and GLP-1RA therapies and perhaps recommendations to referring physicians. Improving access to both endocrinologists and cardiologists for patients with type 2 diabetes and established CVD would seem important given our observations that the therapies that have demonstrated CV benefit are not being readily used in this population, and given the ever-increasing complexity of diabetes and CV-risk management overall. Lastly, we observed that in both patients with and without established CVD, approximately 75% were seen by a primary care provider (IM or family medicine) at least 1 time within the 1-year observation period. In patients with established CVD, approximately 35% of patients were seen ≥ 4 times by a PCP within the same 1-year period. This observation highlights the opportunity that PCPs have to initiate these medications associated with CV risk reduction when appropriate.

The strengths of this study include the large number of participants identified, the use of the validated EMERGE algorithm to properly identify patients with type 2 diabetes, and the robust amount of clinical data which allowed for an extensive depiction of the participants (e.g., calculation of DCSI scores). While there are numerous strengths of our analysis, it is not without limitations. Our study is limited by its use of only structured data to document both CVD and comorbidities (largely by ICD codes), which relies heavily on provider coding practices. Adding the use of natural language processing of chart notes may better capture these disease states vs. using the ICD codes alone. In addition, our record of medication utilization was based upon EHR documentation of prescriptions, not based on pharmacy data; thus medication compliance could not be ascertained. As is typical of cross-sectional data in general, full interpretation of the observed medication use and specialty visit findings is limited by a lack of longitudinal data. Further, cross-sectional data cannot capture the complexities of clinical care on a patient level. However, our intent was primarily to understand the prevalence of use of these medications and overall care patterns in a large cohort of patients with both T2D and CVD, purposes for which cross sectional data are particularly useful. Lastly, our report only contains data from one integrated delivery system from two US regions (Ohio, Florida), so generalizability of our findings may be limited.

## Conclusions

These data provide a real-world assessment of the current treatment landscape and healthcare utilization patterns of patients with type 2 diabetes with established CVD prior to availability of the most recent versions of the ADA and AACE guidelines. These findings highlight that there is certainly room for improvement in terms of prescribing medications that have demonstrated CV benefit to patients who would potentially maximally benefit from such therapies. New guidelines support the need for more holistic diabetes management including consideration of factors beyond simple glycemic control when choosing antidiabetic therapy. The potential clinical impact of the new guidelines remains to be seen and will hopefully be evaluated in the future with additional real-world data.

## Appendix

See Table 4.

**Table 4:** A complete list of all ICD-9/ICD-10 codes for atherosclerotic CVD included in our analysis/definition of established CVD

ADA class	ICD-9		ICD-10	
	430–438	Cerebrovascular disease	I60–I69	Cerebrovascular diseases
Stroke	430	Subarachnoid hemorrhage	I60	Nontraumatic subarachnoid hemorrhage
Stroke	431	Intracerebral hemorrhage	I61	Nontraumatic intracerebral hemorrhage
Stroke	432	Other and unspecified intracranial hemorrhage	I62	Other and unspecified nontraumatic intracranial hemorrhage
Stroke	433	Occlusion and stenosis of precerebral arteries	I63	Cerebral infarction
Stroke	434	Occlusion of cerebral arteries	I65	Occlusion and stenosis of precerebral arteries, not resulting in cerebral infarction
TIA	435	Transient cerebral ischemia		
Stroke	436	Acute, but ill-defined, cerebrovascular disease	I66	Occlusion and stenosis of cerebral arteries, not resulting in cerebral infarction
Stroke	437.0	Cerebral atherosclerosis	I67.2	Cerebral atherosclerosis
Stroke	437.1	Other generalized ischemic cerebrovascular disease	I67.81	Acute cerebrovascular insufficiency
			I67.82	Cerebral ischemia
			I67.83	Posterior reversible encephalopathy syndrome
			I67.84	Cerebral vasospasm and vasoconstriction
Stroke	438	Late effects of cerebrovascular disease	I69	Sequelae of cerebrovascular disease
Stroke			R29.7	National Institutes of Health Stroke Scale (NIHSS) score Exclude R29.700

ADA class	410–414	Ischemic heart disease	I20–I25	Ischemic heart diseases
MI	410	Acute myocardial infarction	I21	ST elevation (STEMI) and non-ST elevation (NSTEMI) myocardial infarction
MI			I22	Subsequent ST elevation (STEMI) and non-ST elevation (NSTEMI) myocardial infarction
ACS	411	Other acute and subacute forms of ischemic heart disease	I24	Other acute ischemic heart diseases
MI	412	Old myocardial infarction	I23	Certain current complications following ST elevation (STEMI) and non-ST elevation (NSTEMI) myocardial infarction (within the 28 day period)
Angina	413	Angina pectoris	I20	Angina pectoris
ACS	414	Other forms of chronic ischemic heart disease Exclude 414.1	I25	Chronic ischemic heart disease Exclude I25.3 and I25.4
ACS	V71.7	Observation for suspected cardiovascular disease		

ADA class	440–449	Diseases of arteries, arterioles, and capillaries	I70–I79	Diseases of arteries, arterioles, and capillaries
PAD	440	Atherosclerosis	I70	Atherosclerosis
PAD	443.8	Other specified peripheral vascular diseases		
PAD	443.9	Peripheral vascular disease, unspecified	I73.9	Peripheral vascular disease, unspecified
PAD	444	Arterial embolism and thrombosis	I74	Arterial embolism and thrombosis
PAD	445	Atheroembolism	I75	Atheroembolism

ADA class		Other CV-related		Other CV-related
MI	429.7	Certain sequelae of myocardial infarction, not elsewhere classified		
TIA			G45	Transient cerebral ischemic attacks and related syndromes
PAD	459.8	Other specified disorders of circulatory system	I99	Other and unspecified disorders of circulatory system
PAD	459.9	Unspecified circulatory system disorder	I99	Other and unspecified disorders of circulatory system
PAD	V12.5	Personal history of diseases of circulatory system	Z86.7	Personal history of diseases of the circulatory system
Revascularization^a^	V45.81	Aortocoronary bypass status	Z95.1	Presence of of aortocoronary bypass graft
Revascularization^a^	V45.82	Percutaneous transluminal coronary angioplasty status	Z98.6	Angioplasty status
Revascularization^a^	V15.1	Personal history of surgery to heart and great vessels, presenting hazards to health	Z95.5	Presence of coronary angioplasty implant and graft
Revascularization^a^			Z95.8	Presence of other cardiac and vascular implants and grafts
Revascularization^a^			Z95.9	Presence of cardiac and vascular implant and graft, unspecified

Per the ADA 2017 guidelines, atherosclerotic CVD was defined as follows: acute coronary syndrome, history of myocardial infarction, stable or unstable angina, coronary or other arterial revascularization, stroke, transient ischemic attack, or peripheral arterial disease presumed to be of atherosclerotic origin

*ACS* acute coronary syndrome, *PAD* peripheral arterial disease, *MI* history of myocardial infarction, *TIA* transient ischemic attack

^a^Revascularization: coronary or other arterial revascularization
